# Electronic and optical properties of chemically modified 2D GaAs nanoribbons

**DOI:** 10.1038/s41598-023-42855-y

**Published:** 2023-09-19

**Authors:** Mahmoud A. S. Sakr, Mohamed A. Saad, Hazem Abdelsalam, Nahed H. Teleb, Qinfang Zhang

**Affiliations:** 1 Chemistry Department, Center of Basic Science (CBS), Misr University of Science and Technology (MUST), 6th October City, Egypt; 2 Physics Department, Center of Basic Science (CBS), Misr University of Science and Technology (MUST), 6th October City, Egypt; 3 School of Materials Science and Engineering, Yancheng Institute of Technology, Yancheng, 224051 People’s Republic of China; 4 Theoretical Physics Department, National Research Centre, El-Buhouth Str., Dokki, Giza, 12622 Egypt; 5 Electron Microscope and Thin Films Department, National Research Centre, El-Buhouth Str., Dokki, Giza, 12622 Egypt

**Keywords:** Chemistry, Materials science, Nanoscience and technology, Optics and photonics, Physics

## Abstract

We employed density functional theory calculations to investigate the electronic and optical characteristics of finite GaAs nanoribbons (NRs). Our study encompasses chemical alterations including doping, functionalization, and complete passivation, aimed at tailoring NR properties. The structural stability of these NRs was affirmed by detecting real vibrational frequencies in infrared spectra, indicating dynamical stability. Positive binding energies further corroborated the robust formation of NRs. Analysis of doped GaAs nanoribbons revealed a diverse range of energy gaps (approximately 2.672 to 5.132 eV). The introduction of F atoms through passivation extended the gap to 5.132 eV, while Cu atoms introduced via edge doping reduced it to 2.672 eV. A density of states analysis indicated that As atom orbitals primarily contributed to occupied molecular orbitals, while Ga atom orbitals significantly influenced unoccupied states. This suggested As atoms as electron donors and Ga atoms as electron acceptors in potential interactions. We investigated excited-state electron–hole interactions through various indices, including electron–hole overlap and charge-transfer length. These insights enriched our understanding of these interactions. Notably, UV–Vis absorption spectra exhibited intriguing phenomena. Doping with Te, Cu, W, and Mo induced redshifts, while functionalization induced red/blue shifts in GaAs-34NR spectra. Passivation, functionalization, and doping collectively enhanced electrical conductivity, highlighting the potential for improving material properties. Among the compounds studied, GaAs-34NR-edg-Cu demonstrated the highest electrical conductivity, while GaAs-34NR displayed the lowest. In summary, our comprehensive investigation offers valuable insights into customizing GaAs nanoribbon characteristics, with promising implications for nanoelectronics and optoelectronics applications.

## Introduction

Two-dimensional (2D) materials have demonstrated exceptional physical and chemical properties, positioning them as top contenders for next-generation technologies^1–4^. These ultrathin and ultralight materials can exist as a single layer, such as graphene^5^, silicene^6^, transition metal dichalcogenides (TMDs)^7^, and mxenes^8^, or as a few layers, including layered graphene^9^, phosphorene^10^, and heterostructures^11^. Their unique characteristics make them highly promising for a wide range of applications, spanning electronics^12^, optoelectronics^13^, spintronics^14^, quantum computing^15^, sensors^16^, catalysis^17^, energy storage^18^, and photovoltaics^19,20^.

Two-dimensional quantum dots (2D-QDs) refer to small-sized materials, typically measuring around 20 nm or less, that possess unique properties influenced by their size and edge characteristics^21–26^. These nanodots offer remarkable control over their electronic and optical properties, as their characteristics can be adjusted by manipulating their size, edge termination, and chemical modifications. Decreasing the size of a material in a quantum dot leads to an increase in its electronic and optical energy gap^27–30^. Furthermore, the application potential of 2D-QDs can be significantly enhanced through chemical functionalization, which involves processes such as doping^31^, introducing vacancies^32,33^, or attaching chemical groups^34–37^. The ability to tailor the physical and chemical properties of these nanodots using these approaches has greatly expanded their range of applications. For instance, hexagonal boron nitride (hBN) quantum dots, when modified with various chemical groups, exhibit a remarkable capacity for detecting metal ions, displaying boosted adsorption energy compared to pristine nanodots^38^. Additionally, chemically modified quantum dots derived from graphene, phosphorene, transition metal dichalcogenides (TMDs), and mxenes have been extensively studied for their potential in sensing and removing diverse pollutants^39–43^. The abundance of active sites, large specific surface area, engineered energy gaps, superior photo-trapping capabilities, and multi-exciton generation make 2D-QDs highly promising for catalytic applications^44^. For example, Mohanty et al. demonstrated that MoS_2_ quantum dots serve as highly efficient catalysts for the oxygen evolution reaction (OER), achieving a low overpotential of approximately 0.37 V^45^. Similarly, antimonene nanoclusters exhibit an even lower overpotential of around 0.31 V for OER at the edges^46^. In the field of photovoltaics, achieving highly efficient solar cells requires three key characteristics. Firstly, there should be effective charge separation on the donor and acceptor layers to facilitate the efficient collection of electrons and holes at the terminals. Secondly, a small conduction band offset is desired. Finally, the donor material should possess a suitable energy gap to facilitate the absorption of incident sunlight. Edge-functionalized phosphorene^47^ and graphene/silicene^48^ quantum dots have shown significant promise in meeting these requirements, displaying notably high power conversion efficiency.

This study investigates the electronic and optical properties of GaAs quantum dots, with a specific focus on finite nanoribbons. The research explores the impact of passivation, functionalization, and doping on these properties. Pristine nanoribbons demonstrate semiconductor behavior with a wide energy gap. However, by introducing specific modifications, the energy gap can be finely tuned. For instance, doping the nanoribbons with Cu atoms at the edge of GaAs nanoribbons reduces the energy gap to 2.672 eV, while full passivation with F atoms increases it to 5.132 eV. These controlled adjustments in the energy gap render GaAs nanoribbons highly adaptable for diverse applications. Moreover, chemical modifications also affect the optical properties of the nanoribbons. The introduction of dopants, passivation, or functionalization at the edges and surfaces of GaAs nanoribbons results in noticeable shifts in their UV–Vis spectra. These shifts can either be a redshift or a blueshift, depending on the nature of the dopant or the specific passivating or functionalizing agent employed. These tunable optical properties further enhance the potential applications of finite GaAs nanoribbons in a wide range of optical and optoelectronic devices.

## Computational model

The structure optimization, electronic, and optical properties are investigated using density functional theory (DFT)^26,37,49–55^ calculations as implemented in Gaussian 16^56,57^. The considered functional is the long-range-corrected WB97XD that yields improved accuracy for non-covalent interactions^58,59^. We also consider the LANL2DZ basis set^60^ that gives acceptable results accuracy at moderate computational power^61,62^. The optical calculations are performed using time-dependent DFT calculations for the first 20 excited states. All the hole and electron parameters, such as the overlap between electron and hole density distributions (Sr index), used to describe the nature of the excited states are calculated using Multiwfn software^63^. The quantum stability chemical (QSC) parameters like dipole moment (μ), chemical potential (ρ), electronegativity (χ), and chemical hardness (η) were calculated using the HOMO energy (E_H_) and the LUMO (E_L_). These QSC parameters are obtained from the following equations $\uprho =\frac{{E}_{H}+ {E}_{L}}{2}$, χ $= -\frac{{E}_{H}+{E}_{L}}{2},\,\mathrm{ and\, \eta }\,=\frac{{E}_{L}-{E}_{H}}{2}$^52,55,64–67^. The binding energy (BE) is calculated from the equation; BE = (N_Ga_E_Ga_ + N_As_E_As_ + N_d_E_d_ + N_p_E_p_ + N_f_E_f_ − E_t_)/N_t_. With N_Ga_, N_As_, and N_t_ are the numbers of Ga, As, and the total number of atoms, respectively. In cases of chemical modifications, N_d_, N_p,_ and N_f_ represent the number of dopants, passivating, and functionalization atoms, respectively. E_Ga_, E_As_, E_d_, E_p_, E_f_, and E_t_ are the corresponding total energies of the Ga, As, d, p, and f atoms, and the final compound, respectively.

To characterize overlapping extent of hole and electron, S_r_ index is defined as follows ${S}_{r}$=$\int {S}_{r}\left(r\right)dr= \int \sqrt{{\rho }^{hole}\left(r\right){\rho }^{ele}\left(r\right)dr}$^68^. Where *⍴*^hole^(r) and *⍴*^ele^(r) is the density of hole and electron particles at specific position r. The total magnitude of charge transfere (CT) length is referred to as^68^ D index: $D \,index =\left|D\right|\equiv \sqrt{{(D}_{x}{)}^{2}+ {(D}_{Y}{)}^{2}+ {(D}_{Z}{)}^{2}}$, where D_X_, D_Y_ and D_Z_ is the magnitude of CT length in in X/Y/Z. H_λ_ measures average degree of spatial extension of hole and electron distribution in the X/Y/Z direction, H_CT_ is in the CT direction, and the H index is an overall measure $H\, index=(\left|{\sigma }_{ele}\right|+ \left|{\sigma }_{hole}\right|)/2$^68^. The |σ_hole_| and |σ_ele_| are referred to as σ_hole_ and σ_ele_ indices, they measure the overall Root Mean Square Deviation (RMSD) of hole and electron, respectively. t index^68^ is designed to measure the separation degree of hole and electron in CT direction: $t\, index=D\, index-{H}_{CT}$. The word Calculate ∆r index to measure charge-transfer length Theory^69^, Δr index was proposed to measure CT length during electron excitation. The Δr can be expressed as $\Delta\, r=\left|\langle {\varphi }_{a}\left|r\right|{\varphi }_{a}\rangle -\langle {\varphi }_{i}\left|r\right|{\varphi }_{i}\rangle \right|\equiv \left|\int r{\left|{\varphi }_{a}(r)\right|}^{2}dr-\int r{\left|{\varphi }_{i}(r)\right|}^{2}dr\right|$. The index i and a run over all occupied and virtual MOs, respectively. φ is an orbital wave function.

## Results and discussion

**Figure 1:**
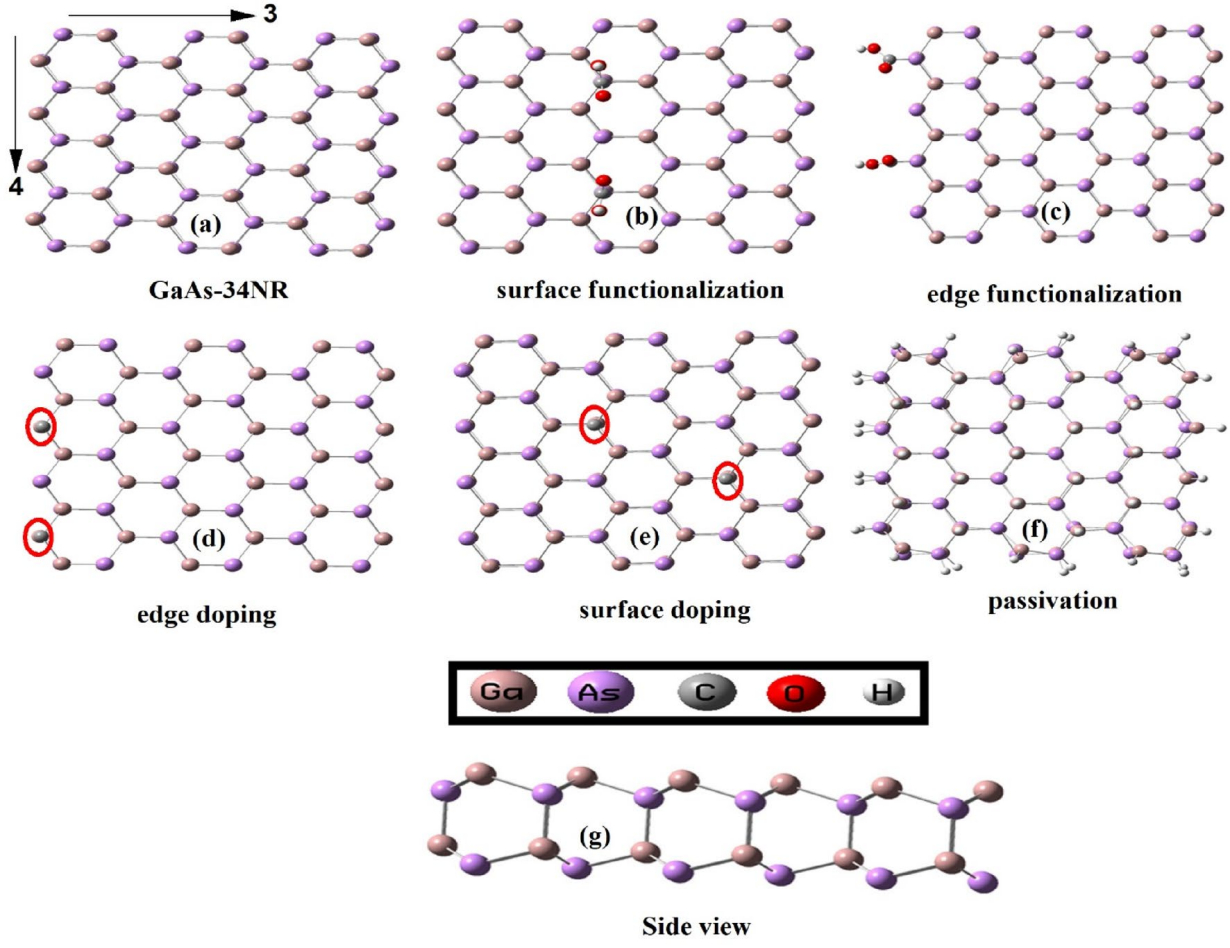
(**a**) GaAs nanoribbons with a 3-armchair edge and 4-zigzag edge. The chemical passivation by hydrogenation (GaAs-34NR-H) is shown in (**f**), edge and surface doping (GaAs-34NR-2C) (**d**, and **e**) as well as edge surface functionalization (GaAs-34NR-2COOH) are shown in (**b**, and **c**) respectively. The side view is shown in (**g**).

Figure 1 illustrates the finite nanoribbons in their original state and following chemical modification. Specifically, the GaAs nanoribbons featuring 3-armchair hexagons and 4-zigzag edge hexagons are referred to as GaAs-34NR, as depicted in Fig. 1a. Subsequently, we conducted additional chemical modifications, including passivation, doping, and functionalization, as demonstrated in Fig. 1b–f. Passivation was applied to cover the surface and the edges of GaAs-34NR. As for doping and functionalization, they were targeted at the edges and surface of GaAs-34NR, as displayed in Fig. 1b–f.

### Structure stability

The stability of the GaAs nanoribbons is investigated by calculating the binding energy and by performing frequency calculations. We included an analysis of the calculated binding energies and the infrared (IR) spectra to confirm the stability and characteristics of the GaAs nanoribbons. The positive binding energies, ranging from 3.315 to 3.646 eV, indicate the stable formation of GaAs nanoribbons, comparable to other two-dimensional quantum dots such as phosphorene and antimonene nanodots^42,70^. We have depicted these values for each structure in Fig. S1 of Supplementary Appendix A. Furthermore, we observed that certain modifications to the nanoribbons affected the binding energy. Passivation with hydrogen and doping with 2W at the edge slightly decreased the binding energy, as shown in Fig. S1d,i. Conversely, doping with two carbon or oxygen atoms and functionalization with the COOH functional group increased the binding energy, as depicted in Fig. S1b–f,h. To further assess the dynamical stability of the structures, we analyzed the IR spectra obtained from frequency calculations. The real vibrational frequencies displayed in Fig. S1 indicate the absence of saddle points on the potential energy surface, confirming the dynamical stability of all considered structures. Moreover, we propose that the IR spectra can serve as a characteristic tool for identifying GaAs nanoribbons in experimental synthesis. The pristine GaAs nanoribbon exhibits vibrational IR peaks at low frequencies, approximately from 300 to 350 cm^−1^, as shown in Fig. S1. However, doping the edge with 2O atoms introduces two additional IR peaks at frequencies of 589.38 and 881.52 cm^−1^. Additionally, passivation with H-atoms introduces two IR peaks at a frequency of ~ 2000 cm^−1^. By including this information in the manuscript, readers will gain insights into the stability and characteristic properties of GaAs nanoribbons, enabling better understanding and identification in experimental settings.

### Electronic properties

#### Pristine nanoribbons and chemical modification by doping

The GaAs-34NR lattice comprises square close-packed Ga and As atoms. Our calculations on the optimized GaAs-34NR structure reveal a significant energy gap of 5.125 eV, attributed to quantum confinement effects. This larger energy gap in GaAs NRs suggests enhanced electron confinement, resulting in discrete electronic states and a greater energy separation between valence and conduction bands. The total density of state (TDOS) and partial density of state (PDOS) spectra provide insights into electronic properties, showing the number of energy states and orbital characteristics^1^. As-atoms acts as electron donor with higher-energy HOMO, primarily composed of 4p orbitals, while Ga-atoms acts as electron acceptor. These findings highlight the unique optoelectronic properties of GaAs NRs compared to bulk counterparts^71^.

**Figure 2:**
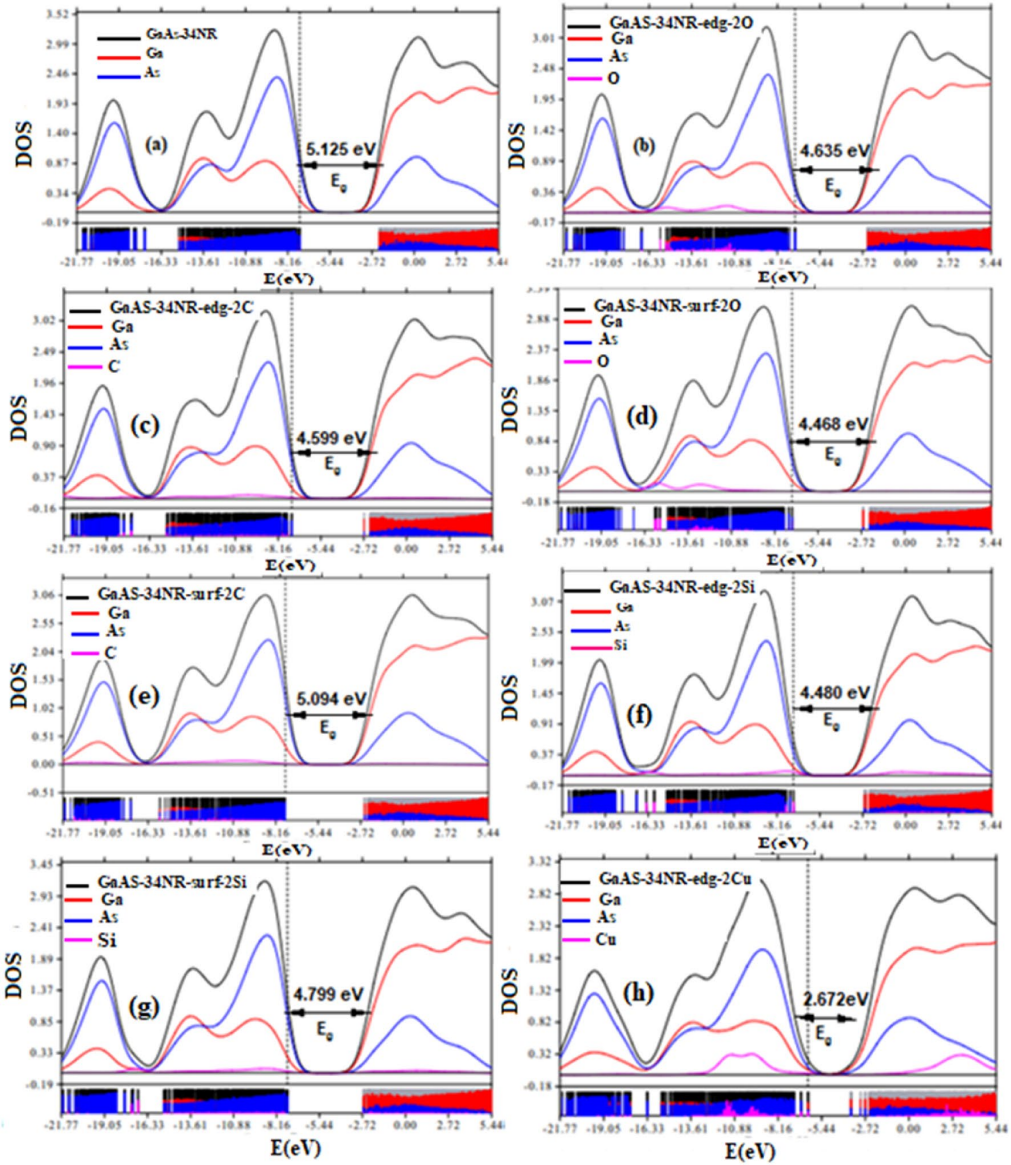
The partial density of states of GaAs-34NR (**a**), GaAs-34NR-edg-2C, GaAs-34NR-surf-2C, GaAs-34NR-edg-2O, GaAs-34NR-surf-2O, GaAs-34NR-edg-2Si, GaAs-34NR-surf-2Si, GaAs-34NR-edg-2Cu (**b–h**).

**Figure 3:**
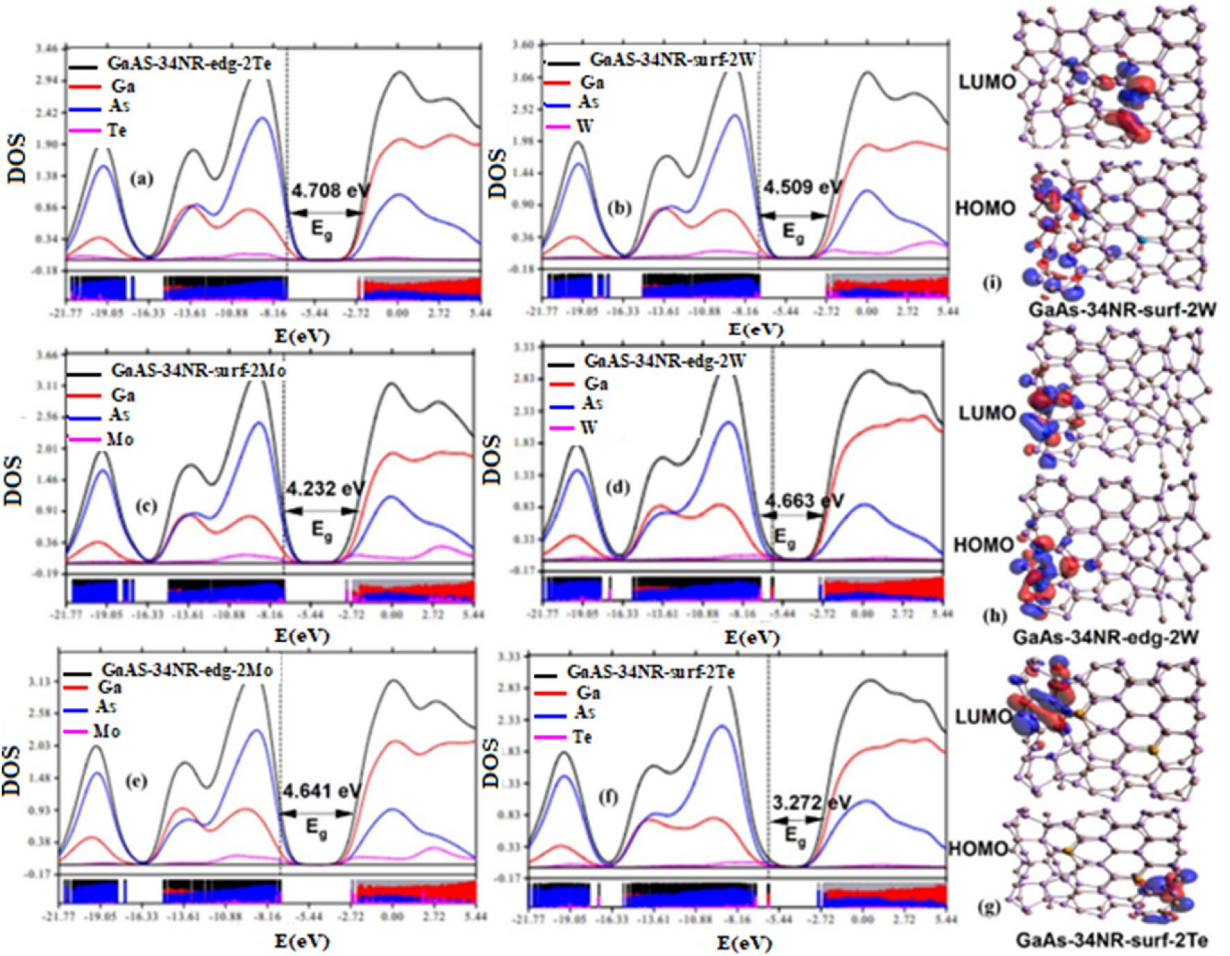
Partial density of states of GaAs-34NR after substitutional doping with 2Te, 2W, and 2Mo (**a–f**) and the corresponding HOMO/LUMO (**g–i**).

The electronic properties of GaAs-34NR and its modifications were investigated using the PDOS and the highest occupied/lowest unoccupied molecular orbitals (HOMO/LUMO). The PDOS analysis was performed using the Multiwfn software^63^, which calculated the percentage contribution of each atom to the molecular orbitals. The results showed that the occupied molecular orbitals were mainly contributed by As-atoms, as indicated by the blue peaks in both GaAs-34NR and its modified structures. Conversely, the unoccupied molecular orbitals were primarily contributed by Ga-atoms, as shown by the red peaks in the PDOS spectra (Figs. 2a–h, 3a–f). This trend was also observed in the HOMO/LUMO distributions presented in Fig. 3g–i and Figs. S2 and S3 of Supplementary Appendix B. Specifically, the HOMO predominantly localized on As-atoms, while the LUMO exhibited an extended distribution along the Ga-Ga bond. The localized distribution of HOMO cubes can be attributed to the lone pair electrons in As-atoms, whereas the LUMO’s extended distribution suggests a strong interaction within the Ga–Ga bond. The manipulation of electronic properties in pristine GaAs nanoribbons was achieved through chemical modifications involving the substitutional doping of 2As-atoms with different elements such as 2C, 2Si, 2Cu, 2O, 2W, 2Te, and 2Mo at both the edge and surface. The doping process had a significant effect on the electronic properties, as evidenced by the PDOS and HOMO/LUMO distributions shown in Figs. 2 and 3. The energy gap of the chemically modified GaAs-34NR structures ranged from 2.672 to 5.094 eV, as observed in Figs. 2 and 3. Interestingly, all the doping atoms at the edge and surface led to a decrease in the energy gap, as depicted in Figs. 2a–h and 3a–f. For example, when 2Cu atoms were doped at the edge and 2Te atoms at the surface, the energy gap of pristine GaAs-34NR reduced from 5.125–2.672 to 3.272 eV, respectively, as shown in Figs. 2h, and 3f. These findings emphasize that GaAs-34NR and its chemically modified derivatives can be employed as semiconductors with varying energy gaps, ranging from tiny to wide or even insulator gaps. The ability to manipulate the electronic properties through chemical modifications opens new possibilities for tailoring the optoelectronic behaviors of GaAs nanoribbons.

#### Passivation and functionalization

**Figure 4:**
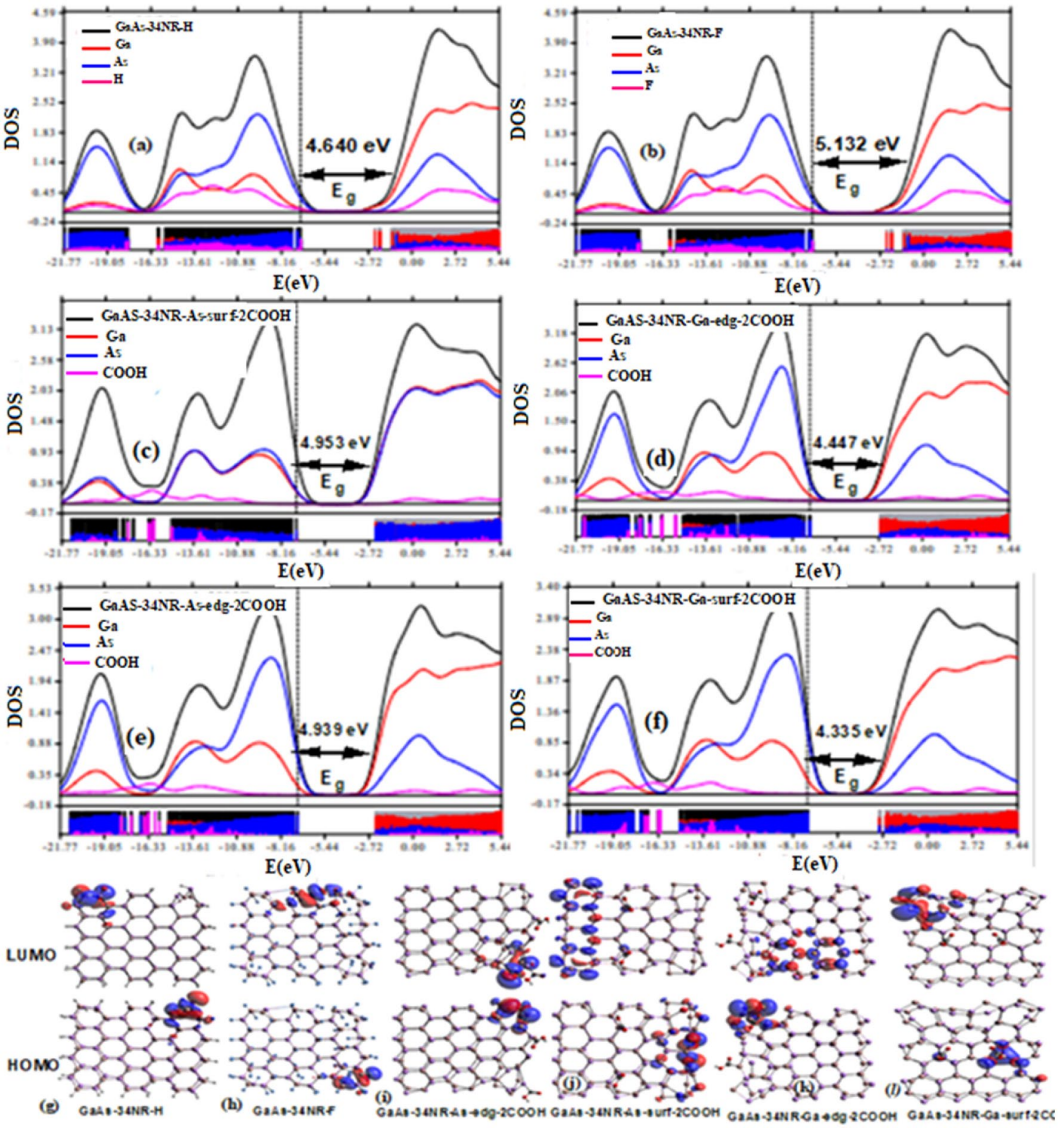
Partial density of states of GaAs-34NR after passivation and functionalization with H, F, and 2COOH (**a–f**) and the related HOMO/LUMO are given in (**g–l**).

Chemical modification plays a crucial role in manipulating the electronic properties of pristine GaAs nanoribbons, either through natural occurrences during experimental synthesis or through deliberate additions. In this study, two types of modifications were considered: (a) functionalization with 2COOH groups at As and Ga atoms on both the edge and surface of GaAs-34NR, and (b) full passivation of Ga and As atoms with H and F atoms. These modifications had a significant impact on the electronic properties, as evidenced by the PDOS and HOMO/LUMO distributions presented in Fig. 4a–l. For instance, passivation with F atoms led to a slight increase in the energy gap of pristine GaAs-34NR from 5.125 to 5.132 eV, as shown in Fig. 4b. On the other hand, hydrogen passivation and functionalization with 2COOH groups at As and Ga atoms on both the edge and surface of the nanoribbons resulted in a decrease in the energy gap. Specifically, the energy gap was reduced to 4.640 eV with hydrogen passivation, and in the range of 4.355 to 4.939 eV with functionalization using 2COOH groups (Fig. 4a,c–f). These findings highlight the effectiveness of passivation and functionalization in tailoring the electronic properties of GaAs nanoribbons. Depending on the specific modifications, the resulting nanoribbons can exhibit wide or even insulator energy gaps, making them suitable for various semiconductor applications.

### Quantum stability

It is worth noting that structures with higher values of μ exhibit an asymmetry in their electronic charge distribution. Among the examined structures, GaAs-34NR-Ga-surf-2COOH displayed the highest magnitude of μ, as indicated in Table 1, followed by GaAs-34NR-edg-Cu. This suggests that GaAs-34NR-Ga-surf-2COOH experiences more active intramolecular charge transfer compared to other structures. Conversely, GaAs-34NR-F exhibited the most negative ρ value compared to other systems, indicating a higher electron escaping tendency. The high electronegativity values for GaAs nanoribbons reflect their strong electron-attracting characteristics. Nanoribbons with F passivation, particularly GaAs-34NR-F, displayed the highest χ value of 7.092 among the structures examined. The introduction of H atoms through passivation reduced χ to 4.641 due to the passivation of Ga and As atoms. Chemical hardness (η), which measures resistance to charge transfer, was found to be highest in GaAs-34NR, indicating its greater resistance to charge transfer compared to other structures. In contrast, GaAs-34NR-edg-Cu exhibited the lowest hardness value, suggesting lower resistance to charge transfer. In conclusion, the reactivity is increased by F passivation, binding 2COOH functional groups to Ga atoms at the surface of the nanoribbons and doping with 2Mo atoms on the surface of GaAs-34NR. Conversely, reactivity is decreased by H passivation, functionalization with 2COOH at the edge and surface, and doping with atoms such as 2O, 2W, 2C, and 2Te (refer to Table 1). Additionally, passivation and doping lead to a decrease in the chemical hardness of GaAs-34NR. F passivation increases electronegativity, while H passivation and all doping atoms at the edge and surface decrease electronegativity.

**Table 1:** The HOMO energy (E_H_), the LUMO energy (E_L_), chemical potential (ρ), electronegativity (χ), chemical hardness (η), dipole moment (μ) and the electrical conductivity (σ) for selected GaAs nanoribbons with and without chemical modification.

Compounds	E_H_ (eV)	E_L_ (eV)	ρ (eV)	χ(eV)	η (eV)	μ (D)	σ (eV)
GaAs-34NR	− 7.930	− 2.265	− 5.098	5.098	2.833	24.111	1.5 × 10^–96^
GaAs-34NR-H	− 6.961	− 2.321	− 4.641	4.641	2.320	33.267	3.3 × 10^–79^
GaAs-34NR-F	− 9.750	− 4.433	− 7.092	7.092	2.659	13.157	1.1 × 10^–90^
GaAs-34NR-As-edg-2COOH	− 7.127	− 2.257	− 4.692	4.692	2.435	29.121	4.3 × 10^–83^
GaAs-34NR-As-surf-2COOH	− 7.224	− 2.271	− 4.748	4.748	2.477	14.610	1.7 × 10^–84^
GaAs-34NR-Ga-edg-2COOH	− 7.006	− 2.619	− 4.813	4.813	2.194	18.030	6.3 × 10^–75^
GaAs-34NR-Ga-surf-2COOH	− 7.285	− 2.930	− 5.108	5.108	2.178	42.322	2.2 × 10^–74^
GaAs-34NR-edg-C	− 7.296	− 2.697	− 4.997	4.997	2.300	17.699	1.6 × 10^–78^
GaAs-34NR-surf-C	− 7.545	− 2.451	− 4.998	4.998	2.547	20.568	7.0 × 10^–87^
GaAs-34NR-edg-O	− 7.030	− 2.395	− 4.713	4.713	2.318	22.531	4.0 × 10^–79^
GaAs-34NR-surf-O	− 7.073	− 2.065	− 4.569	4.569	2.504	18.219	2.0 × 10^–85^
GaAs-34NR-edg-Si	− 7.123	− 2.643	− 4.883	4.883	2.240	20.868	1.7 × 10^–76^
GaAs-34NR-surf-Si	− 7.472	− 2.673	− 5.073	5.073	2.400	24.887	6.8 × 10^–82^
GaAs-34NR-edg-Te	− 7.263	− 2.555	− 4.909	4.909	2.354	23.882	2.3 × 10^–80^
GaAs-34NR-surf-Te	− 6.140	− 2.686	− 4.413	4.413	1.727	35.345	3.8 × 10^–59^
GaAs-34NR-edg-Cu	− 6.294	− 3.622	− 4.958	4.958	1.336	38.992	6.4 × 10^–46^
GaAs-34NR-surf-Cu	− 7.271	− 2.811	− 5.041	5.041	2.230	20.156	3.7 × 10^–76^
GaAs-34NR-edg-W	− 7.159	− 2.496	− 4.828	4.828	2.332	17.581	1.3 × 10^–79^
GaAs-34NR-surf-W	− 7.235	− 2.726	− 4.981	4.981	2.255	11.080	5.5 × 10^–77^
GaAs-34NR-edg-Mo	− 7.303	− 2.662	− 4.983	4.983	2.321	17.356	3.2 × 10^–79^
GaAs-34NR-surf-Mo	− 7.227	− 2.995	− 5.111	5.111	2.116	12.871	2.6 × 10^–72^

GaAs’ wider energy gap suggests improved electron confinement, which leads to more distinct electronic states and a wider energy difference between the valence and conduction bands. The electrical conductivity can therefore be greatly changed by doping, painting, and functionalizing^72–76^. The electrical conductivity analysis of GaAs-34NR (and its derivatives) was performed for electrons using the equation $\sigma =\mathrm{exp}(\frac{{-E}_{g}}{2KT})$^77^. In this equation, σ represents the electrical conductivity, K stands for Boltzmann’s constant, T denotes the thermodynamic temperature, and E_g_ corresponds to the band gap value of the different configurations. A smaller E_g_ value at a given temperature results in higher conductivity. The computed electrical conductivity values have been compiled in Table 1. The findings in Table 1 demonstrate that through passivation, functionalization, and doping processes, the electrical conductivity of GaAs-34NR was enhanced. Notably, among the studied compounds, GaAs-34NR-edg-Cu exhibits the highest electrical conductivity, while GaAs-34NR showcases the lowest conductivity.

### Characterization of excited states

The characterization of excited states is essential for understanding the electronic structure and properties of molecules. Several indices have been developed to describe these states, including the Sr index, D index, t index, and Δr index, which provide information on the overlap between electron and hole densities, centroid coordinates of holes and electrons, degree of separation between holes and electrons, and charge-transfer length, respectively. Figure S4a–t in Supplementary Appendix C displays hole/electron maps, while Table 2 presents data on various indices such as D, Sr, t, Ec, and Δr. To identify representative states, the first excitation state, S1, has been chosen. The Δr index quantifies the charge-transfer length during electron excitation. Transitions from the ground state (S0) to the excited state (S1) in GaAs-34NR-edg-W and GaAs-34NR-edg-Mo primarily exhibit local excitations (LE) based on the significantly low Δr indices. The suggested criterion of 2.0 Å distinguishes between LE and charge-transfer excitations (CT) according to the original paper on Δr^69^. Conversely, the other molecular structures listed in Table 2 display Δr indices higher than 2, indicating the predominance of CT excitations. Fig. S4b,n demonstrates that passivation with hydrogens and doping with 2Cu at the edge result in larger distances between the centers of the hole (blue color) and electron (green color) isosurfaces (Chole and Cele centroids) with corresponding D values of 7.901 and 12.227 Å, respectively, compared to other studied nanoribbon structures. Next, the Sr index is examined, and it is observed that GaAs-34NR-H and GaAs-34NR-edg-2Cu exhibit relatively small Sr indices compared to the other molecular structures, primarily due to their high D index values. Specifically, the S0 → S1 transition of GaAs-34NR-H and GaAs-34NR-edg-2Cu displays a low Sr value of 0.058 and 0.123 au respectively, indicating overlapping between the hole and electron in their S1 excited states, unlike the other studied molecular structures. Regarding the t index, all studied nanoribbons except GaAs-34NR-H, GaAs-34NR-F, GaAs-34NR-edg-O, GaAs-34NR-surf-Te, and GaAs-34NR-edg-Cu exhibit negative values for the excitations from the S0 to the S1 excited state. This suggests a minimal degree of separation between holes and electrons in the S1 state of most nanoribbon structures, except for the aforementioned ones. Conversely, the positive t index values for GaAs-34NR-H, GaAs-34NR-F, GaAs-34NR-edg-O, GaAs-34NR-surf-Te, and GaAs-34NR-edg-Cu indicate a significant degree of separation between holes and electrons in their S1 excited states.

**Table 2:** Charge-transfer length (∆r), centroid coordinates of holes and electrons (D), electron–hole overlap (S_r_), and hole–electron degree of separation (t), for GaAs nanoribbons in S1 excited state.

Nanoribbon	D (Å)	S_r_ (au)	H (Å)	t (Å)	∆r (Å)
GaAs-34NR	1.021	0.421	7.522	− 1.277	2.297
GaAs-34NR-H	7.901	0.123	4.871	4.830	11.661
GaAs-34NR-F	3.152	0.294	4.020	1.208	6.765
GaAs-34NR-As-edg-2COOH	2.244	0.385	4.309	− 0.017	6.639
GaAs-34NR-As-surf-2COOH	0.935	0.398	7.423	− 1.494	2.600
GaAs-34NR-Ga-edg-2COOH	2.706	0.350	4.898	− 0.100	6.682
GaAs-34NR-Ga-surf-2COOH	1.676	0.359	5.097	− 0.671	6.331
GaAs-34NR-edg-C	0.799	0.418	4.680	− 1.681	6.579
GaAs-34NR-surf-C	0.864	0.359	5.418	− 2.208	4.554
GaAs-34NR-edg-O	2.415	0.363	4.387	0.177	6.430
GaAs-34NR-surf-O	0.588	0.380	5.142	− 1.719	3.949
GaAs-34NR-edg-Te	1.283	0.343	7.592	− 3.481	4.398
GaAs-34NR-surf-Te	4.515	0.286	4.326	1.987	9.645
GaAs-34NR-edg-Cu	12.227	0.058	3.964	10.035	12.228
GaAs-34NR-surf-Cu	1.359	0.376	5.050	− 1.586	7.220
GaAs-34NR-edg-W	0.970	0.573	3.857	− 1.876	1.086
GaAs-34NR-surf-W	1.035	0.460	4.369	− 1.860	3.887
GaAs-34NR-edg-Mo	0.766	0.555	4.022	− 1.910	1.268
GaAs-34NR-surf-Mo	1.406	0.433	3.916	− 1.002	5.579

The centroid coordinates of holes and electrons between GaAs-34NR-As-edg-2COOH and GaAs-34NR-As-surf-2COOH are larger due to the specific changes introduced by the different configurations of the nanoribbons. In the case of GaAs-34NR-As-edg-2COOH, the presence of atoms at the edge (As-edg) and the incorporation of 2COOH functional groups promote alterations in the electronic structure of the material. These changes likely influence the distribution of charges within the nanoribbon. The edge atoms and the chemical groups could create energy levels that attract or repel electrons and holes, shifting their positions and leading to larger centroid coordinates. This effect could be a result of electronic interactions between the edge atoms and the functional groups, causing charges to spread out more. On the other hand, in GaAs-34NR-As-surf-2COOH, the absence of edge atoms and the different positioning of the functional groups might lead to less pronounced changes in the charge distribution, resulting in smaller centroid coordinates for holes and electrons.

### Optical properties

**Figure 5:**
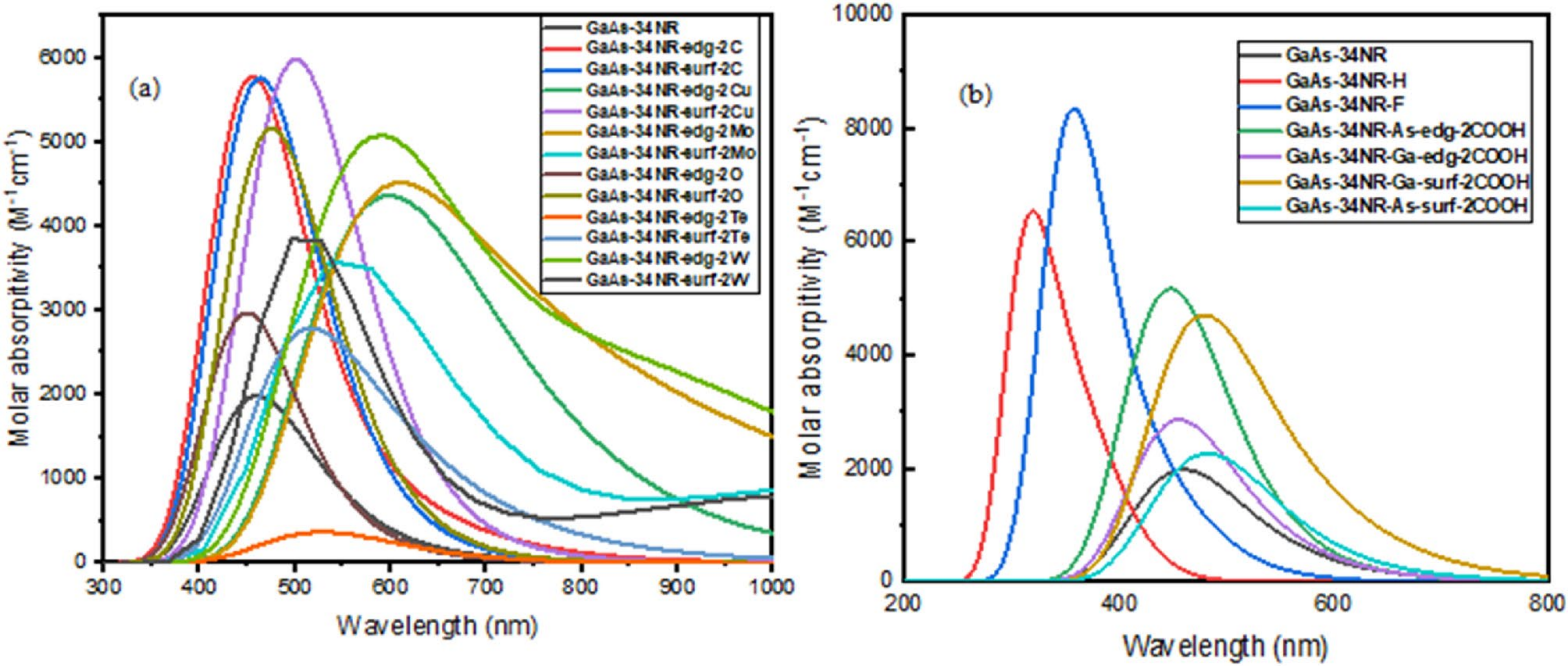
(**a**) The UV–Vis absorption spectra of GaAs-34NR before and after modification by doping with different elements at both edge and surface. (**b**) The absorption spectra of GaAs-34NR before and after passivation with H- and F-atoms and functionalization with the COOH.

In this section, we focused on studying the UV–Vis absorption spectra of various GaAs nanoribbons: GaAs-34NR, GaAs-34NR-H, GaAs-34NR-F, GaAs-34NR-edg-2C, GaAs-34NR-surf-2C, GaAs-34NR-edg-2Cu, GaAs-34NR-surf-2Cu, GaAs-34NR-edg-2W, GaAs-34NR-surf-2W, GaAs-34NR-edg-2Te, GaAs-34NR-surf-2Te, GaAs-34NR-edg-2O, GaAs-34NR-surf-2O, GaAs-34NR-edg-2Mo, GaAs-34NR-surf-2Mo, GaAs-34NR-As-edg-2COOH, GaAs-34NR-As-surf-2COOH, GaAs-34NR-Ga-edg-2COOH, and GaAs-34NR-Ga-surf-2COOH. We have presented the resulting spectra in Fig. 5, and the relevant parameters can be found in Table 3. The primary objective of this investigation was to analyze the impact of passivation with hydrogen and fluoride, as well as doping with carbon, oxygen, copper, tungsten, trillium, and molybdenum, on both the edge and surface of the GaAs-34NR structure in terms of UV–Vis absorption spectra. By the electronic properties, the absorption spectra originate from electronic transitions between occupied and unoccupied molecular orbitals (MOs). Thus, the effects observed in the optical absorption peaks due to size and chemical modifications should align with the aforementioned electronic effects. The introduction of passivation, doping, and functionalization significantly influences the absorption spectra of GaAs-34NR, resulting in either a redshift or a blueshift, as illustrated in Fig. 5a,b. For instance, passivating GaAs-34NR with hydrogen and fluoride atoms induces a blueshift in the absorption spectrum. In particular, λ_max_ shifts to 320.630 and 356.380 nm, respectively, due to H → L + 1 and H-4 → L + 4 transitions. Similarly, doping with 2C (at both the edge and surface) and 2O (at the surface) leads to a blueshift towards lower wavelengths, resulting in absorption peaks at 455.840, 458.850, and 451.020 nm, respectively. These observations are in agreement with the behavior of the electronic energy gap after passivation and doping. On the other hand, doping with 2Te, 2Cu, 2W, and 2Mo (at both the edge and surface) causes a redshift in the absorption spectra (refer to Table 3). Additionally, when 2COOH functional groups are linked to As and Ga atoms at the surface of the nanoribbon, a redshift is observed. However, when the same functional groups are linked at the edge, a blueshift occurs. This discrepancy arises because the primary optical transition is now defined by the energy difference between specific states, such as H → L + 14, H-1 → L + 2, H-3 → L + 4, and H-7 → L, rather than the traditional H → L transition as observed in the ideal case of the electronic energy gap. By incorporating these findings into the manuscript, we provide a comprehensive analysis of the influence of various modifications on the UV–Vis absorption spectra of GaAs-34NR, highlighting the shifts in absorption peaks and their correspondence to electronic energy changes.

**Table 3:** The calculated excited state (ES), maximum wavelength (λ_max_), transition energy (TE), electronic transition (ET), oscillator strength (*f*), and transition coefficient (TC) for GaAs-34NR and its modified materials.

Nanoribbon	ES	λ_max_	TE (eV)	ET	*f*	TC
GaAs-34NR	14	462.150	2.682	H → L + 3	0.0089	0.110
GaAs-34NR-H	15	320.630	3.866	H → L + 1	0.0018	0.491
GaAs-34NR-F	13	356.380	3.479	H-4 → L + 4	0.0106	0.106
GaAs-34NR-As-edg-2COOH	12	447.940	2.768	H → L + 14	0.0080	0.106
GaAs-34NR-As-surf-2COOH	11	481.940	2.573	H-1 → L + 2	0.0032	0.421
GaAs-34NR-Ga-edg-2COOH	12	456.790	2.725	H-3 → L + 4	0.0075	0.136
GaAs-34NR-Ga-surf-2COOH	10	480.250	2.582	H-7 → L	0.0014	0.150
GaAs-34NR-edg-C	14	455.840	2.720	H-1 → L	0.0071	0.179
GaAs-34NR-surf-C	13	458.850	2.702	H-1 → L + 5	0.0128	0.115
GaAs-34NR-edg-O	12	451.020	2.749	H → L + 16	0.0076	0.101
GaAs-34NR-surf-O	13	473.780	2.617	H → L + 4	0.0078	0.169
GaAs-34NR-edg-Te	3	535.130	2.317	H → L + 3	0.0006	0.226
GaAs-34NR-surf-Te	10	512.450	2.420	H → L	0.0021	0.107
GaAs-34NR-edg-Cu	8	599.700	2.067	H → L + 2	0.0030	0.618
GaAs-34NR-surf-Cu	12	496.650	2.496	H-4 → L + 2	0.0228	0.113
GaAs-34NR-edg-W	10	581.920	2.131	H-2 → L + 15	0.0162	0.280
GaAs-34NR-surf-W	12	531.950	2.331	H → L + 5	0.0063	0.121
GaAs-34NR-edg-Mo	11	609.060	2.036	H → L + 11	0.0083	0.147
GaAs-34NR-surf-Mo	10	578.250	2.144	H → L + 7	0.0022	0.175

## Conclusion

In this study, we investigated the structure stability, electronic properties, and optical properties of finite GaAs nanoribbons using density functional theory calculations. We considered various chemical modifications, including passivation, doping, and functionalization, to understand their effects on the nanoribbons. To ensure the stability of the structures, two key factors were examined. Firstly, the positive binding energy confirmed their stability, indicating that the formation of GaAs nanoribbons was energetically favorable. Additionally, real vibrational frequencies observed in the infrared absorption spectra provided further evidence of their stability. The investigated nanoribbons are characterized by a wide energy gap that could be precisely tuned through chemical functionalization. The energy gap ranged from 2.672 to 5.094 eV, demonstrating the potential for fine-tuning the electronic properties of GaAs nanoribbons. For instance, passivating the edges and surfaces of GaAs nanoribbons with F atoms significantly increased the energy gap to approximately 5.132 eV. Conversely, doping the pristine GaAs nanoribbon with Cu atoms at the edge (GaAs-34NR) reduced the energy gap to 2.672 eV. Analyzing the partial density of states, we observed that the density peaks corresponding to occupied orbitals were predominantly contributed by As-orbitals, whereas the unoccupied orbitals were dominated by Ga atomic orbitals. This indicated that arsenide atoms acted as electron-donating sites, while Ga atoms acted as electron-accepting sites. The distribution of the highest occupied and lowest unoccupied molecular orbitals further supported these findings. To gain insights into the electron–hole interactions in excited states, we calculated various indices such as the charge-transfer length (∆r) and electron–hole overlaying (Sr). These indices provided valuable information about the nature of electron–hole interactions within the system. The UV–Vis absorption spectra exhibited a redshift when doping with 2Te, 2Cu, 2W, and 2Mo (at both the edge and surface), indicating a shift towards longer wavelengths. Furthermore, chemical functionalization played a significant role in modulating the absorption spectra of GaAs-34NR, leading to either a redshift or a blueshift depending on the dopant site or the attached element.

### Supplementary Information

Supplementary Information.

## Data Availability

All data generated or analyzed during this study are included in this published article.
