# Supplementary material for: Electronic and optical properties of chemically modified 2D GaAs nanoribbons
Source: Sci Rep. 2023 Sep 19;13:15535. doi: 10.1038/s41598-023-42855-y (PMC10509254; doi:10.1038/s41598-023-42855-y)
Supplement: Supplementary file 1 — Supplementary Information. [file 41598_2023_42855_MOESM1_ESM.docx]

**Appendix A**

The infrared (IR) spectra of the pristine and chemically modified GaAs finite nanoribbons are shown here. It is observed that all the considered structures have positive vibrational frequencies responsible for the IR absorption peaks. It is also observed that pristine GaAs nanoribbons have several IR absorption peaks distributing at low frequencies ~ from 250-300 cm^-1^. The chemical modification introduces additional IR peaks at higher frequencies, for instance, the additional two peaks appear around 2250 cm^-1^ after passivation with H-atoms, see Fig. S1 (i).


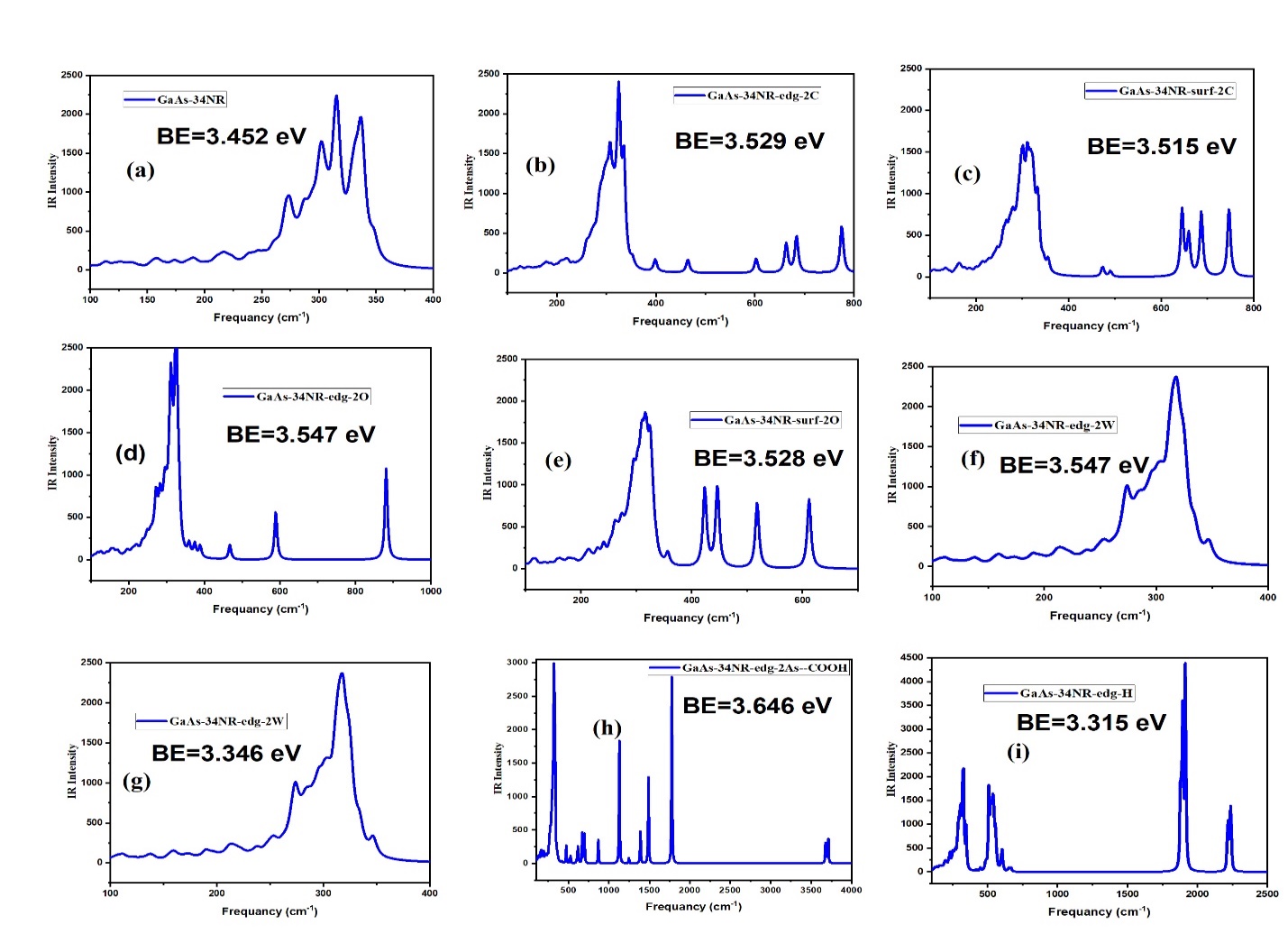


**Fig. S1** (a) The infrared spectra of GaAs nanoribbons. (b-i) The IR spectra of the selected chemically modified structure.

**Appendix B**

**
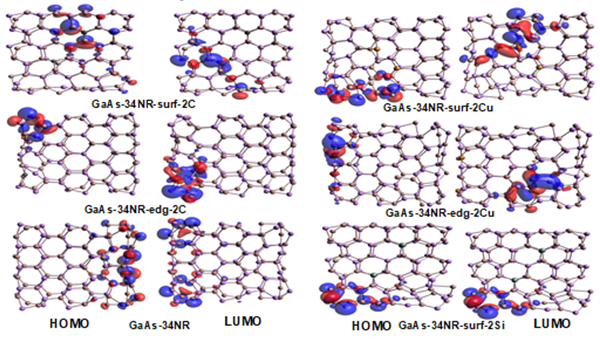
**

Fig.S2 HOMO and LUMO of GaAs-34NR after substitution with 2C, 2Cu at both edge and surface also for GaAS-34NR-surf-2Si.


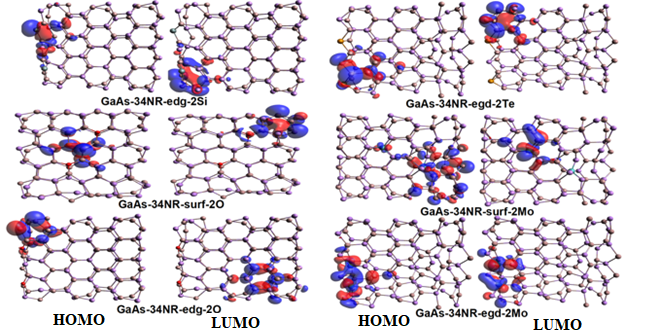


Fig.S3 HOMO and LUMO of GaAs-34NR-edg-2Si, GaAs-34NR-edge-2Te, GaAs-34NR-surf-2O, GaAs-34NR-edg-2O, GaAs-34NR-surf-2Mo and GaAs-34NR-edg-2Mo

**Appendix C**


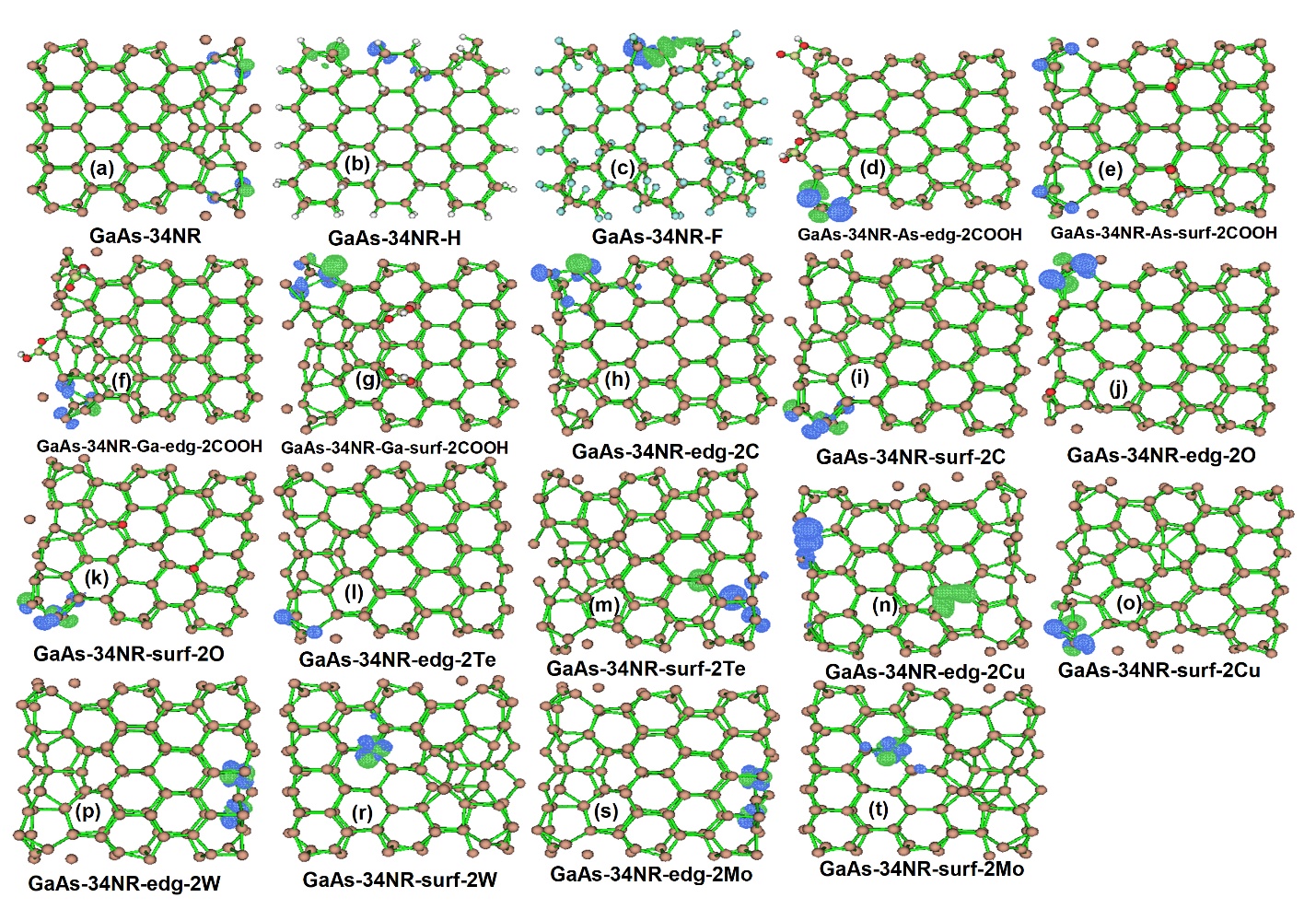


Fig. S4hole/electron maps of GaAs-34NR and its modified molecular structures.
